# Emerging Role of the Spleen in the Pharmacokinetics of Monoclonal Antibodies, Nanoparticles and Exosomes

**DOI:** 10.3390/ijms18061249

**Published:** 2017-06-10

**Authors:** Mauro Cataldi, Chiara Vigliotti, Teresa Mosca, MariaRosaria Cammarota, Domenico Capone

**Affiliations:** 1 Division of Pharmacology, Department of Neuroscience, Reproductive Sciences and Dentistry, Federico II University of Naples, 80131 Naples, Italy; chiara86.vigliotti@gmail.com (C.V.); mariaro.cammarota@gmail.com (M.C.); 2 Section of Clinical Pharmacology, Integrated Care Department of Clinical Neurosciences, Anesthesiology and Drug-Use, Federico II University Hospital, 80131 Naples, Italy; teresa.mosca@unina.it (T.M.); docapone@unina.it (D.C.)

**Keywords:** spleen, monoclonal antibodies, nanoparticles, exosomes, accelerated blood clearance, marginal zone

## Abstract

After being absorbed, drugs distribute in the body in part to reach target tissues, in part to be disposed in tissues where they do not exert clinically-relevant effects. Therapeutically-relevant effects are usually terminated by drug metabolism and/or elimination. The role that has been traditionally ascribed to the spleen in these fundamental pharmacokinetic processes was definitely marginal. However, due to its high blood flow and to the characteristics of its microcirculation, this organ would be expected to be significantly exposed to large, new generation drugs that can hardly penetrate in other tissues with tight endothelial barriers. In the present review, we examine the involvement of the spleen in the disposition of monoclonal antibodies, nanoparticles and exosomes and the possible implications for their therapeutic efficacy and toxicity. The data that we will review lead to the conclusion that a new role is emerging for the spleen in the pharmacokinetics of new generation drugs, hence suggesting that this small, neglected organ will certainly deserve stronger attention by pharmacologists in the future.

## 1. Introduction

“What does that do a spleen?” asked Charles Freck to Jim Barris in the famous novel “A Scanner Darkly” by Philip K. Dick (1977), and this is the question that probably most of us would ask if we were told that spleen could have something to do with drug action or disposition. The spleen is, indeed, barely mentioned in pharmacology textbooks, and among the physiological roles of this organ, such as immunological surveillance, removal of aged blood cells, hematopoiesis and the regulation of blood volume [1], neither drug disposition, nor the involvement in pharmacological drug action are ever mentioned. The little attention paid to the spleen in pharmacology was probably due to the lack of evidence that it could have a major role in the disposition of the “classical” drugs. However, as often happens when a new character appears in a novel and the perspective on the story drastically changes, also our way of looking at the relationship between drugs and the spleen is now changing as a consequence of the development of “new-generation” drugs. With the generic term of “new-generation” drugs, we intend here to refer not only to really new drugs, such as to nanoparticle drugs (e.g., liposomes or nanotubes), but also to drugs that are not so new anymore, such as biotechnological drugs (e.g., recombinant proteins and monoclonal antibodies). “New” generation drugs differ from “classical” drugs for the much more complex chemical structure and for their larger size, which make them more similar to the antigen particles to which spleen physiologically responds than to traditional drugs. In the present review, we will go through the evidence showing that the spleen may affect the disposition of monoclonal antibodies, nanoparticles and exosomes not only contributing to their clearance, but also representing, in selected cases, an important target organ where their pharmacological effects are exerted. Before addressing these points, we first have to examine the microanatomy characteristics of the spleen that set the structural basis for drug-spleen interaction.

## 2. Spleen Microanatomy: The Pharmacologist Point of View

**Figure 1:**
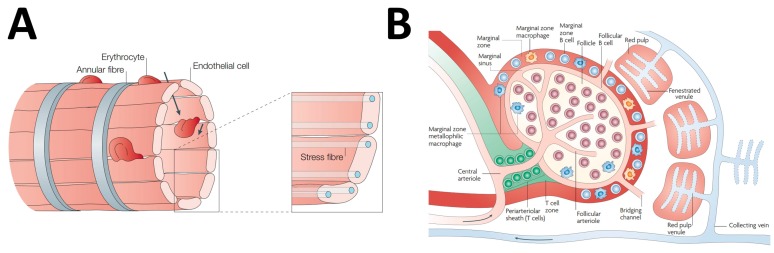
Microanatomy of the spleen circulation. (**A**) Structure of the splenic venous sinuses. The drawing shows how venous sinuses of the splenic red pulp are delimited by endothelial cells arranged in a barrel-like fashion making narrow slits that erythrocytes have to cross to return into the venous circulation. As detailed in the text, aged or damaged cells that are too rigid to cross these slits are retained in the red pulp and phagocytosed by macrophages. Reproduced from [1] with permission from the publisher; (**B**) Structure of the microcirculation of the spleen. The drawing schematically illustrates how small vessels emerging from the penicillary artery form the marginal sinuses at the boundary between white and red pulp. The marginal zone with its specific cell populations such as metallophilic macrophages is represented, as well. Notice that in this pictorial representation, a model of open splenic circulation was adopted with penicillary arteries freely opening in red pulp. Reproduced from [15] with permission from the publisher.

The spleen has a very special microanatomy that makes it very intriguing from a pharmacological point of view. A systematic analysis of splenic structure is beyond our aims, and the interested readers can find details on this issue in textbooks and in several excellent reviews [2,3,4,5,6]. Here, we will explore the more salient aspects that give very specific properties to this organ from the perspective of drug diffusion and, possibly, metabolism and action. We will address this point by starting with the analysis of how the vasculature distributes inside the spleen because this is the easiest way to describe the microanatomy of this complex organ. Once that the lineal artery enters the spleen through the hilus of the spleen, it branches in arteries of progressively smaller caliber that run inside the fibrous trabeculae originating from the splenic capsule till their end. Then, they become central arteries that are surrounded by sheaths of lymphatic tissue often enlarging to form splenic follicles making up the so-called white pulp. After exiting from the white pulp, the central arteries further branch to form the penicillar arteries that enter the red pulp of the spleen. Whereas the white pulp of the spleen is essentially made of lymphatic tissue, there are two main kinds of structures in the red pulp: the sinuses and the splenic cords [7]. Splenic sinuses are unique structures different from conventional capillaries. They are essentially cavities lined up by a discontinuous endothelium. Endothelial cells are disposed in parallel, hence delimiting narrow slits that represent the anatomical site where the sieving activity of the spleen is exerted (Figure 1A). Splenic sinuses continue in venules that anastomose to form veins of progressively larger caliber. Splenic cords are cavities inside the stroma of the red pulp that are filled up by red and white cells. Splenic cords are not lined by endothelium, but represent a specialization of the stroma and, as such, are delimited by fibroblasts and extracellular matrix. Red blood cells in splenic cords have to squeeze across the splits of the splenic sinusoids to return to the general circulation (Figure 1A). Aged and diseased erythrocytes that are not deformable enough to cross these slits are retained in the red pulp and destroyed [8]. As we will discuss later, this is also a mechanism involved in nanoparticle sequestration by the spleen. There has been a long controversy on how the splenic sinuses and cords are fed by the penicillary arteries in human spleen. For a long time, the prevalent model has been that penicillary arteries open up in the red pulp with no direct continuity with the wall of sinuses, hence making an open circulatory system in which blood may freely exit from the arterial compartment [9,10]. Further studies suggested instead that penicillary arteries directly continue in sinuses (closed circulation models), and combined models in which both open and closed circulation coexist were proposed, as well. In 2011, Steiniger et al. reported a detailed 3D reconstruction of red pulp vessels of the human spleen showing that virtually all of the circulation was of an open type [7]. Furthermore, another area of controversy concerning the blood supply of the white pulp has been recently addressed by 3D reconstruction studies. Kusumi et al. [11] showed that different from what was suggested by previous evidence, the central arteries do not directly contribute to the vascularization of the white pulp. The white pulp is, instead, irrorated by harpin loop arterioles emerging from the penicillary artery; after that, it leaves the follicle or the lymphoid sheath surrounding the central artery. An important contribution also comes from the central arteries of neighbor follicles. The arterioles that emerge from the penicillary arteries and those that come from neighbor follicles give rise to discontinuous capillary-like structures, the *marginal sinuses*, at the junction between the red and the white pulp (Figure 1B). This region known as the marginal zone (MZ) is well defined in rats and mice, but not in humans [5]. MZ is populated by resident IgM+/IgD− memory B lymphocytes, and it is easily identifiable in histological preparations, being demarcated by a uniform population of metallophilic macrophages (Figure 1B). A second population of macrophages, the MZ macrophages, is scattered throughout the entire MZ among lymphocytes. Both MZ metallophilic macrophages and MZ macrophages express high levels of sialoadhesin, an adhesion molecule belonging to the Sialic acid-binding ImmunoGlobulin-type LECtins (SIGLEC) family [12]. The structure of the MZ is well suited for triggering early antibody response to blood-borne antigens because these molecules may freely cross the marginal sinus and have access to macrophages that act as antigen-presenting cells to memory B lymphocytes. The structure of human white pulp is quite different from that of rodents because no clear MZ is observed at the junction of white and red pulp. Instead, a specialized structure known as the perifollicular zone has been identified in humans [13]. This zone is viewed as an extension of the sinuses of the red zone, as it is directly connected with this structure. As the rodent MZ, it contains also sheathed capillaries and a population of sialoadhesin positive cells, but different from rodents, not only IgM+/IgD−, but also IgD+ lymphocytes are commonly observed in the perifollicular zone [14].

Because of the structural characteristics that we quickly reviewed, the splenic microcirculation is fully permeable to blood-borne molecules. This property is potentially relevant from a pharmacokinetics point of view considering that it sets the premises for a significant distribution inside the splenic parenchyma also of drugs that are excluded from the majority of the other organs either because of their size or of their charge. As we will discuss with some detail later, this is especially relevant for large “new generation” drugs such as monoclonal antibodies, large proteins, liposomes and nanoparticles. Assuming that, because of these characteristics of splenic microcirculation, drugs may have free access to the splenic parenchyma, the obvious question is what will be their fate thereafter. Will they be rapidly cleared off or will they be captured and somehow “processed” inside the spleen? Furthermore, how important will be what happens in the spleen for the general fate of the drug in the body? Many of these questions remain still unresolved, and in the present review, we will go through the few data that are available at the moment. Just to start with some general considerations, we will recall that in a system such as the spleen where there is few or no sieving by the endothelial barrier, the amount of drug that will enter into the parenchyma will be critically dependent on how much drug enters into the spleen with the blood, i.e., on splenic blood flow. Splenic blood flow has been quantified with several techniques in the past including ultrasonography also with microbubbles, serial measurement of radioactivity in the splenic area with a scintillation counter after the inhalation of radioactive gases such as ^133^X, capture of ^111^I-labeled platelets or measurement with PET of positron emission after the intravenous injection of H_2_^15^O [16,17,18,19,20]. These studies agree that the spleen has a very high blood flow ranging around 170 mL/min/100 g, making it one of the most perfused organs in the body. It has, however, to be mentioned that because of its small weight, the spleen only receives about 4.8 ± 1.5% of cardiac output [21]; this clearly reduces the potential impact of the spleen in the general economy of the process of drug distribution inside the body. As described above, after entering the spleen, blood can be directed to two different destinations: it can perfuse the white pulp through the marginal or the perifollicular zone, or it can enter the red pulp and go through the splenic cords [22]. In physiological conditions, more than 90% of the splenic blood flow goes through the white pulp bypassing the red pulp [22]. This finding not only highlights the prevalent role of spleen as a lymphoid organ, but also suggests that blood-borne drugs will be predominantly directed to the white pulp once that they enter the spleen, and this has relevant pharmacological implications for several reasons. First, the rate of blood flow significantly slows down when entering the white pulp at the MZ (or perifollicular zone in humans), and this has the extremely relevant consequence of significantly increasing the persistence of blood-borne antigens (but also drugs) at the MZ facilitating the interaction with macrophages and lymphocytes and their penetration though conduits into the deeper white pulp [23]. We will come back to this point later on in the text to explain how it profoundly affects the pharmacokinetics of nanoparticles. The blood filtered through splenic capillaries goes out the spleen with the lymph. Very little is known about lymphatic flow through the spleen, but it appears important not only to maintain convective flow through splenic capillaries, but also because it represents a path for the efflux of activated lymphocytes and released cytokines from the spleen to the general circulation [24].

Considering that the majority of splenic blood flow is directed to the white pulp, the cell types that will be preferentially exposed to drugs coming into the splenic parenchyma from the blood will be lymphocytes and macrophages. Drug interaction with these two cell types may have implications both in terms of therapeutic or toxic activity and in terms of drug disposition. Macrophages and lymphocytes are, indeed, target of clinically-relevant drug classes. For instance, fluoroquinolones and clofazimine accumulate inside macrophages, and 6-mercaptopurine, cyclosporine and saquinavir-ritonavir act on lymphocytes [25,26,27,28,29]. Much less is known about the possible involvement of macrophages and lymphocytes in drug metabolism. It has been clearly demonstrated, however, that macrophages express at very high levels a full array of drug transporters including concentrative nucleoside transporter (CNT) 3, equilibrative nucleoside transporter 3, monocarboxylate transporter (MCT) 1, MCT4, peptide/histidine transporter (PHT) 1, PHT2, organic anion transporting polypeptide transporter 2B1 and ABC pumps multidrug resistance protein (MRP) 1/ABCC1 and MRP3/ABCC3 [30]. These transporters may have a role in intracellular transport of drugs acting on macrophages. Macrophages also express drug-metabolizing enzymes of the cytochrome P450 superfamily [31], and evidence has been reported of drug metabolism by macrophages [32,33]. Similarly, the expression of both drug transporters and drug metabolizing enzymes has been demonstrated in lymphocytes [34,35]. All of these data suggest that the spleen may capture and process classical drugs. This issue, however, did not receive any significant attention in the literature. Conversely, other mechanisms such as the internalization of opsonized small particles are emerging as primarily involved in the disposition of “new generation” drugs. In the next sections, we will go through these other mechanisms by exploring in some detail the role of the spleen in the pharmacology of monoclonal antibodies, nanoparticles and exosomes.

## 3. Role of the Spleen in the Disposition of Monoclonal Antibodies

Monoclonal antibodies are immunoglobulins that are produced form a single cell clone and as such have a very high specificity being directed against a single epitope [36]. Monoclonal antibodies are produced with different technologies and bear different degrees of similarities with human immunoglobulins [36,37]. Besides highly specific antigen recognition that depends on their variable regions, additional pharmacological properties such as opsonization or complement fixation are conferred to these molecules by their constant Fc residues depending on the IgG class to which they belong [37]. Because of their selectivity and specificity, monoclonal antibodies are an important tool in the clinics being able to neutralize and/or trigger the immune destruction of very specific antigen targets. At the beginning of 2017, more than sixty monoclonal antibodies were marketed for human use (http://www.accessdata.fda.gov/scripts/cder/daf/index.cfm?event=BasicSearch.process).

Monoclonal antibodies are highly polar molecules, and their molecular weight ranges around 150 kDa [36,38]. Because of these characteristics, they significantly differ from a pharmacokinetic point of view from “classical” drugs, and their distribution behavior is considered more similar to that of bacterial antigens or nanoparticles. More specifically, while in the case of “classical” drugs, a large part of drug distribution across the endothelial barriers depends on simple diffusion, this process is considered negligible in the case of monoclonal antibodies that penetrate into peripheral tissues, mainly by convection [38]. This process is mainly driven by the differences in hydrostatic pressure between capillary lumen and the interstitium, and as such, it depends on lymph efflux from the interstitium itself. Convection bypasses the plasma-membranes of the endothelial cells as it takes place through the intercellular space, and therefore, it is becomes more and more favored as the intercellular junctions become looser and looser. While moving by convection through the intercellular space, monoclonal antibodies, as any other protein, undergo a process of filtration. This sieving is operated by the connective tissue of the lamina basalis and of the deeper layers of the capillary wall and by the glycocalyx that oppose the transfer of proteins through the capillary wall [39,40]. The process of convective filtration through the capillary wall can be mathematically modeled using the classical Starling equation or its more recent revisions. This equation includes a specific parameter, the reflective coefficient, to account for the different leakiness of the intercellular sieve in the different capillary beds [40]. While in most tissues, capillary barriers have reflective coefficients ranging around 0.95−0.98 and are almost impermeable to plasma proteins, the spleen capillaries are much looser, and accordingly, their reflective coefficients have been estimated to range around 0.85 [41]. This finding is in agreement with the evidence that splenic circulation is freely permeable to plasma proteins [42]. Therefore, a larger amount of monoclonal antibodies is expected to enter into the parenchyma of spleen than in any other organ. This opens the question of what could be their fate thereafter. Very simplistically, we could state that part of them will simply go through the parenchyma and be removed by lymph, and part will be captured by the spleen either to be processed or to be returned unmodified to the blood. Therefore, a deeper understanding of the role of spleen in the pharmacokinetics of monoclonal antibodies requires that these different processes are better characterized and quantified in the context of the whole body disposition of these drugs. Unfortunately, the data available to address this point are still limited. What is clear is that a basic distinction has to be done between monoclonal antibodies that can bind specifically to antigens expressed in cells populating the spleen, and monoclonal antibodies that are directed, instead, against targets that are not present at a significant level in this organ. In the first case, the monoclonal antibody will bind to its target, and the spleen will represent a preferential site of its accumulation and pharmacological action, whereas in the second case, less specific mechanisms of antibody capture will be involved. More specifically, immunoglobulins are cleared from the circulation in a way that is independent from antigen recognition through the interaction with the Fcγ receptors in macrophages, monocytes and neutrophils to be internalized and destroyed [43]. This clearance mechanism is opposed by the immunoglobulin recycling through the FcRn receptors [43]. Because immunoglobulin binding to these receptors is reversible upon acidification, after being internalized, antibodies are released from FcRn in the acidic endosomal compartment and recycled to the plasma [44]. Therefore, FcRns critically control the circulating half-life of immunoglobulins [45,46] that can be increased by targeted mutations of the FcRn interaction site [47]. Importantly, FcRn receptors are also expressed in the spleen where they could limit immunoglobulin degradation by splenic macrophages [48]. This hypothesis is confirmed by data obtained in FcRn knockout mice showing that, in the absence of the FcRn receptors, the liver and the spleen are the most important sites for immunoglobulin accumulation, whereas in control wild-type mice, similar values of tissue accumulation -measured as area under the curve from day 0 to day 7 (AUC_0–7_) of percentage of injected dose per gram of tissue (%ID/g)—are observed in all of the organs [49].

Rituximab is probably the best example of a monoclonal antibody that targets specific splenic antigens, and the analysis of its disposition will make evident how important is the spleen in tissue distribution of this group of monoclonal antibodies. Rituximab binds to CD20 antigens that are expressed in normal late-preB and B cells, including B-cell splenocytes and in neoplastic B lymphoma cells [50]. Moreover, whereas bone marrow and lymph node plasma cells do not express CD20, a significant expression of this antigen has been reported in a subpopulation of short-lived plasma cells that reside in splenic white pulp [51]. Interestingly, these splenic plasma cells may produce autoantibodies and be involved in autoimmune diseases [51]. Consistent with the ability of rituximab to bind to B-cells and plasma cells in this organ, high absorbed doses were observed in the spleen after the administration of radioactive derivatives of this antibody such as ^131^I-Tositumomab or ^99m^Tc-rituximab, indicating a significant splenic accumulation [52,53]. Importantly, in the spleen, bound rituximab exerts its pharmacological effects on target cells by inducing a profound depletion of splenic B-cells [54]. This effect could account for therapeutic effects of this monoclonal antibody both as an antineoplastic drug in splenic localization of B-cell neoplasms [55,56] and as an immunosuppressive agent in organ transplantation recipients [57,58], as well as in patients with autoimmune diseases, such as autoimmune thrombocytopenic purpura and systemic lupus erythematosus [59,60,61,62,63]. The relevance of rituximab distribution in the spleen for its pharmacological effect as an immunosuppressant agent is highlighted by the evidence that long-lived CD20^+^ plasma cells have been identified in the spleen of patients with autoimmune thrombocytopenic purpura or with primary warm auto-immune hemolytic anemia that are resistant to this monoclonal antibody [64,65]. This suggests that when rituximab does not deplete plasma cells in the spleen, it is therapeutically ineffective. Although a detailed analysis of rituximab pharmacodynamics in the spleen goes beyond the aim of this review, we would like to mention that this monoclonal antibody could act in a much more complicated way than simply depleting splenic B-cell. There is evidence, indeed, that by inducing important alterations in the white pulp microenvironment, it could affect also T-cell function and the process of selection of auto-reactive B-cells [66,67,68].

**Figure 2:**
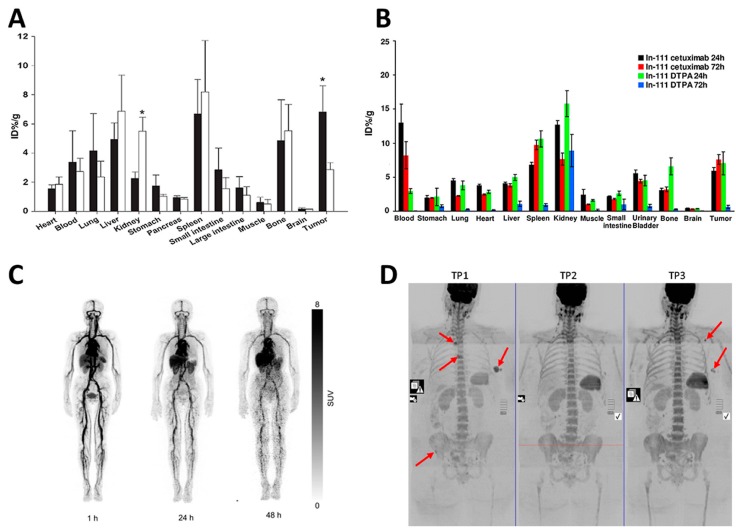
Distribution of monoclonal antibodies into the spleen. (**A**) Tissue distribution of ^89^Zr-bevacizumab (black bars) and of ^89^Zr-IgG (white bars) measured ex vivo as the percent of the injected dose per gram of weight (%ID/g) in different mouse organs collected 168 h after injection. * *p* ≤ 0.05 vs. ^89^Zr-IgG.Reproduced from [70] with open permission; (**B**) Distribution of ^111^In-cetuximab and of diethylenetriaminepentaacetic acid (DTPA) measured ex vivo as %ID/g in organs and in metastatic colorectal tumor radioactivity levels 72 h after the injection. Reproduced from [71] under a Creative Commons Attribution 3.0 License; (**C**) Whole body PET images obtained 1, 24 and 48 h after the injection of ^64^Cu-1,4,7,10-tetraazacyclododecane-1,4,7,10-tetraacetic acid (DOTA)-trastuzumab in a patient with HER2-positive breast cancer. Notice the strong accumulation of the labeled antibody in the spleen at all of the time points. Reproduced under a Creative Commons Attribution License from [86]; (**D**) Splenic enlargement in a patient receiving trastuzumab emtansine for the treatment of metastatic breast cancer. Whole body MRI images obtained in a 59-year-old patient with metastatic breast cancer at baseline (time point 1: TP1) and after eight (time point 2: TP2) and 12 cycles (time point 3: TP3) of treatment with trastuzumab emtansine. Notice the progressive enlargement of the spleen whose estimated volume increased from 92.3 cm^3^ at baseline up to 214.9 cm^3^ at TP3. The arrows indicate the primary breat cancer and bone metastases. Reproduced from [78] with permission from the publisher.

While antigen binding is an obvious mechanism of selective accumulation in the spleen of monoclonal antibodies directed against splenic antigens, the role of this organ in the disposition of monoclonal antibodies that do not directly target splenic antigens is less clear. Studies performed with antibodies labeled with radioactive probes showed that the spleen is a preferential site also for the accumulation of these monoclonal antibodies. For a correct interpretation of studies involving radioactive antibodies, it is important to remember that different radiotracers have different retention times in the tissues where they are captured. Some radiotracers like ^131^I are released into the bloodstream as soon as they are released from the degraded monoclonal antibody, whereas others such as ^111^In accumulate inside the degrading cells: The former will give us a general idea on instantaneous and the latter on cumulative tissue capture (and degradation) of the antibody [69]. Just to mention some important examples of studies evaluating the splenic accumulation of radioactive monoclonal antibodies, Nagengast et al. [70] labeled the anti-VEGF monoclonal antibody bevacizumab either with the long-lived PET isotope ^89^Zr or with ^111^In and followed with a micro PET apparatus its distribution in vivo in nude mice bearing SKOV-3 ovarian tumor xenografts (Figure 2A). They found that whereas during the first 24 h after injection the tracer distributed in animal body with a pattern that paralleled blood perfusion, after 72 h, it was preferentially located in the xenotumor, in the liver and in the spleen. Spleen was also a preferential site of radioactivity accumulation of the anti-HER1-antibody ^111^I-cetuximab in mice bearing subcutaneous HCT-116 colorectal tumor xenografts [71] and of the anti-epidermal growth factor receptor 3 monoclonal antibody ^89^Zr-RG7116 in mice injected with human NSCLC or head and neck carcinoma cell lines [72] (Figure 2B). A significant accumulation in the spleen was also observed for trastuzumab, an anti-HER2 antibody that has a crucial role in the treatment of HER2/neu-positive breast cancer in women [73] (Figure 2C). Remarkably, among all body tissues, the spleen was second only to the blood in the percentage of the injected radioactive dose (%ID/g) that was retained per gram of tissue [73]. The prevalent accumulation of radioactivity in spleen was also observed with ^212^Pb-trastuzumab in mice with orthotopic prostate cancer (PC-3MM2) implantation [74]. Because of the recent enormous impact of this class of immune check point inhibitors in cancer therapy, it is important to mention that a significant splenic accumulation has been recently documented also for two anti-PD-L1 antibodies, ^131^I-labeled MPDL3280A and its derivative PRO304397 [75].

As a whole, the results of the studies with radiolabeled monoclonal antibodies showed that they significantly distribute inside the spleen reaching in this organ concentrations higher than in the majority of the other body organs. Being directed against antigens that are not specifically expressed in the spleen, these monoclonal antibodies are not expected to have any direct impact on splenic function. Drug-conjugated monoclonal antibodies (ADCs) may represent an important exception to this rule. These monoclonal antibodies bear conjugated drugs, usually antitumor chemotherapeutics that are released upon internalization in target cells where they may exert their therapeutic or toxic effects [76]. Under many respects, the pharmacokinetics of ADCs is dominated by the antibody component, and therefore, they are expected to accumulate, be degraded and release their conjugated drugs in the spleen [76]. This could explain why splenic toxicity with hypertrophy and vacuolation of reticuloendothelial system (RES) cells and necrosis and degeneration of lymphocytes have been observed both in rats and in cynomolgus monkeys in preclinical studies with the trastuzumab emtansine conjugate also known as Trastuzumab-DM1 T-DM1 [77]. Interestingly, splenic enlargement has been recently observed at magnetic resonance imaging (MRI) in 92% of the patients treated with T-DM1 for metastatic breast cancer [78] (Figure 2D). Under these premises, the issue of splenic distribution of ADCs and of its possible impact on splenic function will certainly deserve further attention with the larger use in therapy of these drugs [79].

Whereas the data reviewed so far convincingly show that monoclonal antibodies may accumulate in the spleen by the interaction either with specific splenic antigens or with unspecific Ig clearance mechanisms, they do not clarify how important splenic capture is for whole body disposition of these molecules. This point received little attention in the past, and most of the information nowadays available comes from physiologically-based pharmacokinetic (PBPK) modeling studies. In PBPK models, the body is described as composed by a definite number of compartments each of which contributes to drug disposition in a way that can be predicted on the basis of known or estimated physiological parameters such as afferent blood flow or venous return [80]. The first PBPK models of monoclonal antibody disposition did not include FcRn receptors. Using a similar approach, Covell et al. [81] estimated that the spleen is the third most important site for monoclonal antibody degradation after the gut and the liver contributing to 3.6% of whole body disposal. In more recent PBPK models such as the model developed by Garg and Balthasar [82], also FcRn receptors were included. When the role of FcRn was considered, the spleen only marginally contributed to monoclonal antibody catabolism that was mainly operated by skin, muscle, liver and gut (33, 24, 16 and 12% of the total, respectively) [82]. The main reason for the limited involvement of the spleen in the process was that, whilst being highly perfused, this organ receives only a minor fraction of cardiac output, because of its small size. Indeed, in normal adults, it weighs on average 139 g (range, 43–344 g), which represents only about 0.18 of total body weight [83], and it receives about 4.8 + 1.5% of total cardiac output [21]. A predominant role of skin and muscle in the catabolism of monoclonal antibodies was also found by Ferl et al. [84] whose model included FcRn, but was not validated in FcRn-ko mice. More recently, Shah and Betts [85] published a new PBPK model intended to serve as a platform for the pharmacokinetic analysis of new monoclonal antibodies. Different from Garg and Balthasar [82] who gave the same value of 0.95 to vascular reflection coefficient in all organs, Shah and Betts [41] used the data of Sarin [85] on paracellular pore size to calculate the reflection coefficients in different tissues and came out with the values of 0.95 for lung, heart, muscle, skin, adipose, 0.9 for kidney, thymus, small intestine and pancreas, 0.85 for the spleen and 0.84 and for tumors [41]. Despite this substantial change in parameters used for modeling that is expected to increase the estimate of the splenic uptake of monoclonal antibodies, the calculated contribution of the spleen remained low. The model predicted, indeed, a value for the antibody distribution coefficient (i.e., the proportionality constant between blood and tissue concentration) of about 12.8 for the spleen, very close to that of other organs, such as the skin, the gut or the liver, where it ranged between 4% and 16% [41]. Given the similar values of the distribution coefficient, monoclonal antibodies will be expected to accumulate in different organs in amounts proportional to organ weight, and therefore, splenic contribution will be small.

In conclusion, monoclonal antibodies may accumulate in the spleen either by interacting with specific antigens, as in the case of rituximab, or being specifically captured by splenic macrophages. Because of its small size and of the fact that, consequently, it receives only a small fraction of cardiac output, the spleen does not play a major role in monoclonal antibody disposition with the only possible exception of antibodies not interacting with FcRn receptors. However, splenic localization of monoclonal antibodies may cause an important local effect in this organ especially in the case of ADCs.

## 4. Role of the Spleen in the Pharmacokinetics of Nanoparticle Drugs

The progress in the field of nanotechnology opened the way toward the development of new generation drugs differing from classical drugs because of their dimensions in the nanoscale range. Although still very small, these particles are several orders of magnitude larger than classical drugs and represent an important advancement in drug therapy because they can be assembled as multimolecular complexes including not only pharmacologically-active molecules, but also molecules for selective targeting to specific tissues. Therefore, with the help of nanotechnology, conventional drugs, biotechnological and nucleic acid drugs can be incorporated into nanoparticles for selective targeting to specific tissues and to increase their half-life. According to the International Union of Pure and Applied Chemistry (IUPAC), the term “nanoparticles” applies to particles with a size in the range of 10–100 nm [87]. However, as acknowledged by the FDA in its “Guidance for Industry on Considering whether an FDA-regulated product involves the application of nanotechnology” (2004) (https://www.fda.gov/regulatoryinformation/guidances/ucm257698.htm#_ftn10), particles for use in human therapy larger than 100 nm may sometimes have functional characteristics similar to nanoparticles and be considered bona fide nanotechnological drugs. Nanoparticle structure is highly heterogeneous and encompasses entities as different as small lipid vesicles, like liposomes, or semiconductors, like quantum dots. Since the early days of nanoparticle development, it appeared clear that the spleen had an important role in their pharmacokinetics. It was found that unmodified nanoparticles disappear from the blood in seconds or minutes after their injection. Renal filtration has a significant role in this process if their size is smaller than 15 nm, whereas for nanoparticles larger than 40 nm, disappearance from the blood is mainly dependent on their accumulation in cells of the RES [88,89,90]. This significantly reduces nanoparticle half-life and represents a major barrier for the implementation of their clinical use. Moreover, it can lead to phagocyte activation and trigger an inflammatory response that can be responsible of unwanted effects and toxicity [91]. Nanoparticle uptake is mainly operated by liver Kupffer cells, but splenic macrophages also have a significant role [89,92,93,94,95,96]. It is noteworthy that the mechanisms responsible for nanoparticle removal from blood are similar for nanoparticles with very different structures such as liposomes and polymer nanoparticles [97]. The first and most important among these mechanisms is the internalization of opsonized nanoparticles. Opsonization is due to the deposition on nanoparticle surface of opsonins, a heterogeneous group of proteins or protein fragments including C3, C4 and C5 and immunoglobulins, fibronectin and apolipoproteins [98,99] that interact with a number of different surface receptors on RES cells including complement, Fc and fibronectin receptors [100]. Opsonins are not the only plasma proteins that accumulate on the nanoparticle surface. A heterogeneous group of other plasma proteins summing up to tens or hundreds may get loosely bound to nanoparticles’ surface, making up a protein corona that can be recognized by scavenger receptors [97,101]. This represents an additional mechanism responsible for nanoparticle clearance by RES. There has been a certain interest in determining precisely where nanoparticle uptake does take place in the spleen. Using slices of living splenic tissue to test ex vivo the ability of splenic cells to capture nanoparticle, Demoy et al. [102] showed that MZ macrophages can internalize polystyrene nanoparticles and that this internalization process does not involve lectin-like receptors, but scavenger receptors and the albumin coating of the nanoparticles. Furthermore small liposomes (100–200 nm) tend to be selectively internalized by MZ macrophages. This tendency to accumulate in MZ macrophages has been exploited in studies on spleen physiology as a tool to selectively deplete this cell population by using clodronate-loaded liposomes [103,104,105].

**Figure 3:**
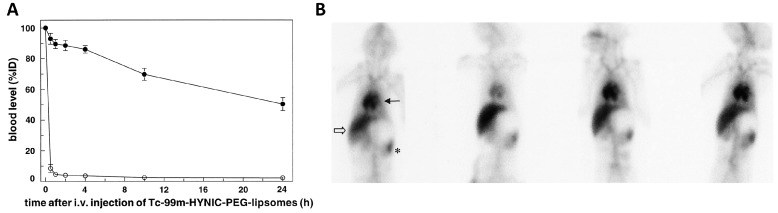
Accelerated blood clearance (ABC) of ^99m^Tc-labeled HYNIC-PEG liposomes. (**A**) Time course of the blood levels of ^99m^Tc-labeled *N*-hydroxysuccinimidyl hydrazino nicotinate hydrochloride (HYNIC)-PEG liposomes measured as the percentage of the injected dose at different times after two consecutive intravenous injections performed in rats with a one-week interval. Notice that whereas blood radioactivity declined slowly after the first injection (black circles), it decreased very rapidly after the second (white circles), hence showing the accelerated blood clearance phenomenon. Reproduced with slight modifications from [116] with permission from the publisher; (**B**) Whole body scintigraphic images obtained in a rhesus monkey after four consecutive intravenous injections of ^99m^Tc-labeled HYNIC-PEG liposomes performed on Day 0, Day 7, Day 21 and Day 35 of the study as indicated. Each image was obtained four hours after each intravenous injection of labeled liposomes. Notice that the spleen is strongly labeled at all time points and that signal intensity over the liver area strongly increases at the second injection performed seven days after the first one. → indicates the heart, ⇒ the liver, and ∗ the spleen region. Reproduced from [116] with permission from the publisher.

Capture by MZ macrophages is not the only fate that nanoparticles can have in the spleen. Indeed, it has been demonstrated that particles larger than 100–200 nm, being incapable of crossing the endothelial slit of splenic sinuses, can be filtered off and retained in the red pulp where they are internalized by red pulp macrophages and slowly destroyed [106,107]. By one hour from the injection, almost all of the large nanoparticles are in the red pulp outside macrophages, whereas they are found inside macrophages by 4 h after being administered [106]. Particle removal by splenic filtration tends to increase with size and is maximal for particles larger than 400 nm [95,102,107,108]. Interestingly, as nanoparticle size increases, Kupffer cell capture decreases, and splenic capture is enhanced. As we will discuss later, it has been proposed that the tendency of larger nanoparticles to be retained in splenic red pulp could represent the basis to develop splenotropic agent for diagnostic or therapeutic purposes. In conclusion, very small (15<nm) unmodified nanoparticles are filtered by the kidney; nanoparticles larger than 15 nm and smaller than 200 nm are captured by Kupffer cells and splenic MZ macrophages; whereas particles larger than 200 nm are retained in the red pulp of the spleen [107]. Therefore, a first strategy that is to be used to minimize splenic capture of nanoparticles and increase their circulating half-life consists of keeping their size small, usually in a size range between 100 and 200 nm. In addition, specific strategies have been developed to prevent nanoparticle interaction with the macrophagic receptors involved in their recognition and capture. To this aim, the nanoparticle surface was covered with different kinds of stealth coating [109]. Surface PEGylation remains the most widely-used of these strategies [110], although a number of alternative stealth coatings based on other polymeric compounds such as poly-*N*-vinylpyrrolidone, poly(amino acid)s, or poly[*N*-(2-hydroxypropyl)methacrylamide] have been also developed [111,112]. An enormous amount of experimental work has been performed to optimize stealth coating with the aim, for instance, of obtaining intelligent nanoparticles whose stealth does not hamper tissue penetration in tumors [113,114]. The analysis of this issue goes beyond the scope of this review, and we will just focus on the “splenic” side of the story by emphasizing that the original expectations that, by stealth coating and a careful control of nanoparticle size, all of the problems related to splenic capture would have been solved, were partially betrayed. It was indeed observed that upon repeated administration, the half-life of nanoparticles progressively decreases [115]. This phenomenon was called accelerated blood clearance (ABC), and as we will discuss later, spleen has an important role in its genesis. The first report on ABC was by Dams et al. [116] who, more than 15 years ago, observed a progressive decrease in the half-life of ^99m^Tc-PEG liposomes after repeated administration in rats and monkeys (Figure 3). Thereafter, ABC was observed with other PEGylated liposome preparations [117,118] and, also, with other non-liposomal PEG-containing-nanoparticles, such as microemulsions, polymeric micelles, polymeric nanoparticles and PEGylated proteins [117].

In the seminal paper by Dams et al. [116], it was observed that ABC can be transferred through serum transfusion from animals repeatedly exposed to PEGylated nanoparticles to never treated animals. It was also observed that the serum factor involved was a protein with a molecular weight of about 150 kD whose identity remained, however, undetermined. Based on the observation that anti-PEG IgM are produced in animals exposed to PEG-conjugated proteins and contribute to their clearance [119,120], Ishida et al. proposed that also the ABC phenomenon could be dependent on IgM generation [121]. By Western blot analysis and HPLC-MS/MS, they showed that the main proteins from the serum of animals that developed ABC that were able to bind liposomes in vitro were anti-PEG IgM [121]. Importantly, upon binding to the liposome surface, these immunoglobulins could also activate the complement, hence providing a mechanism for clearance by macrophages. These observations led to a model that assumed that the ABC consists in a first induction phase that takes place after the first challenges with the nanoparticle and during which IgM are produced, and of a second, effectuation phase that occurs at the following challenges when IgM bind to nanoparticle, activate the complement and promote opsonization by Kupffer cells in the liver [122].

**Figure 4:**
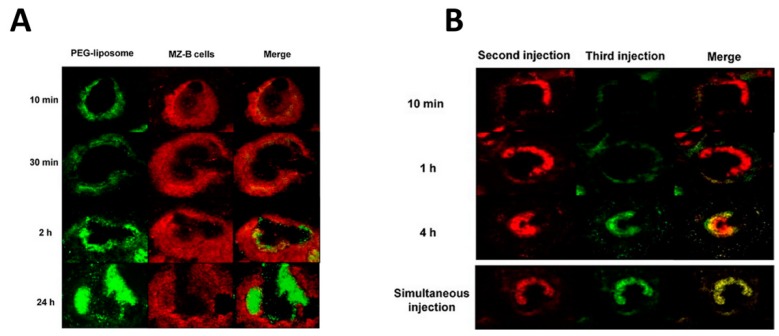
Kinetics of nanoparticle diffusion into the spleen during the accelerated blood clearance (ABC) phenomenon. (**A**) Migration of PEGylated liposomes from the marginal to the follicular zone during the accelerated blood clearance (ABC) phenomenon. The left part of the panel shows fluorescence images obtained 10 min, 30 min, 2 h and 24 h after the injection of fluorescent green PEGylated liposomes in rats in which ABC had been induced by previous administration of non-fluorescent PEGylated liposomes. The middle of the panel shows in the red marginal zone macrophages at the same times, whereas on the right, the merging of the green and red images is reported. Notice that green fluorescence moves from the marginal into the follicular zone where it is almost completely located after 24 h; (**B**) PEGylated liposomes injected at different times independently migrate from the marginal to the follicular zone. After inducing ABC with the injection of a first dose of PEGylated liposomes, a second and a third dose were injected four hours, one apart from the other. To independently track liposomes from the two injections, red and green fluorescent liposomes were used for the second and for the third injection, respectively. Images in the right and in the middle of the panel were obtained 10 min, 1 h and 4 h from the injection of the second and of the third dose as indicated, whereas, on the right, the merging of these images is reported. Notice that both after the second and after the third dose, fluorescent liposomes migrate from the marginal to the follicular zone. In addition, merged images show that when green liposomes from the third injection start accumulating into the marginal zone, those from the second injection (red) are already in the follicular zone. This suggests that the migration process is quick and that liposomes from different injections migrate independently inside splenic white pulp. Images in the bottom of the panel show that when red and green PEGylated liposomes are injected simultaneously, they migrate at the same time from the marginal into the follicular zone. (**A**,**B**) are both reproduced from [127] with permission from the publisher.

The role of the spleen in ABC was firmly established by the evidence that this process can be prevented if animals are splenectomized [123]. Splenectomy is effective till three days after the challenge with the PEGylated nanoparticles and becomes ineffective thereafter. This suggests that the spleen takes part in the induction phase of ABC. In agreement with this hypothesis, it was also observed that upon exposure to PEGylated nanoparticles, IgM concentrations in plasma do not increase in splenectomized animals as in control animals and that their concentration on the surface of liposomes is also lower [123]. These findings suggested that the spleen could be responsible for the IgM response to PEGylated nanoparticles playing a role similar to its involvement in immunological surveillance against blood-borne microorganisms and parasites that elicit a first, rapid IgM response before triggering a more persistent immune response. Several lines of evidence supported the conclusion that ABC induction was due to a T-cell independent activation of MZ B lymphocytes. It was observed, indeed, that the ABC response induced by empty PEG-liposomes was still present in athymic animals and in BALB/c nu/nu mice that lack T-cells, whereas it was impaired in animals whose MZ B lymphocytes had been depleted with cyclophosphamide and in BALB/c SCID mice that lack both T- and B-cells [124,125]. In addition, empty PEG-liposomes failed to induce splenic T-cell proliferation in vitro [124], whereas B lymphocytes isolated from the spleen of animals showing the ABC phenomenon released anti-PEG IgM when challenged in vitro with PEGylated liposomes [117]. This splenic IgM response can be, therefore, classified as a T-independent type 2 response (TI-2) [126]. Using fluorescent PEGylated liposomes, the intrasplenic distribution of these particles was followed over time, and it was observed that immediately after the first injection, they preferentially localize inside MZ B-cells, whereas they are absent in the follicular zone of the white pulp [127]. However, quite early, these liposome-carrying B lymphocytes started to migrate from the MZ to enter the follicular zone passing through the marginal sinus (Figure 4A). Fluorescence was already appreciable in the follicular zone two hours after injection, and after 24 h, it was totally localized in this region and completely absent from the MZ [127] (Figure 4B).

Whereas the ABC phenomenon cannot represent a problem when the use of nanoparticle drugs is planned for a very short time, it could be a major obstacle in the case of long-lasting therapies involving multiple administrations [117]. The case of tumor therapy with nanoparticle carrying chemotherapeutic drugs is especially exemplificative in this perspective. Nanoparticles can be used both in the context of classical chemotherapy protocols involving drug administration at high dosages in short cycles or they can be given as a tool for metronomic therapies that involve repeated administration of low dosages for long times. In the first case, ABC could hardly represent a problem also considering that once those nanoparticles have been internalized in splenic macrophages, the chemotherapeutic drug that they carry may exert its immunosuppressive activity and halt the development of the ABC phenomenon itself. This has been demonstrated for doxorubicin [128] that has been used as a PEGylated liposomal form for more than 20 years with no clinical evidence of tolerance developing by ABC [129]. Conversely, a significant ABC phenomenon was observed with the continued repeated administration of nanoparticles containing low doses of several chemotherapeutic drugs, such as doxorubicin [130], epirubicin [118] or topotecan [131].

The ability of chemotherapeutic drugs to suppress the ABC phenomenon when given at high doses could be used as a tool to prevent its development in therapeutic protocols involving the repeated administration of PEGylated nanoparticles. For instance, it has been shown that when a first high-dose doxorubicin nanoparticle administration is given before the repeated administration of low dose doxorubicin-containing nanoparticles, ABC is totally prevented [132]. Similarly, it has been demonstrated that oxaliplatin containing PEGylated liposomes prevent the production of anti-PEG IgM and the consequent ABC response in the so-called SOXL (S1-OXaliplatinum-Liposomes) regimen, a chemotherapy protocol based on the combined use of metronomic S1 and PEGylated oxaliplatin-containing liposomes [132]. Additional strategies have been developed in the effort of minimizing the ABC phenomenon with mixed results such as the use of poly-glycerol, of zwitterionic poly(carboxybetaine), or of other polymeric stealth coatings instead of PEG, or of red blood cell membrane as a biomimetic nanocoating [113,133,134,135].

Splenic capture can be used to selectively deliver old drugs to the spleen. The evidence that we reviewed so far suggests that spleen capture is a limiting factor in nanoparticle pharmacokinetics and that it has to be overridden in order to optimize their therapeutic effects. However, before closing this section, we want to mention that in selected circumstances, the tendency of nanoparticles to be captured by the RES may actually represent a significant advantage when a selective activity in splenic macrophages or in Kupffer cells is desired. In this perspective, drugs can be loaded in nanoparticles to modify their pharmacokinetic profile and deliberately increase their clearance by splenic macrophages [136]. Polycyanoacrylate nanoparticles were among the first nanoparticles proposed for splenic targeting [137], and they were used to deliver ampicillin into the cytoplasm of macrophages. Because of its poor intracellular distribution, this antibiotic has a limited efficacy against intracellular pathogens that could be increased upon incorporation in nanoparticles. This was demonstrated with ampicillin-loaded polyisohexylcyanoacrylate (PIHCA) nanoparticles in experimental infections in mice with *Listeria monocytogenes* or *Salmonella typhimurium* [138,139]. Similar results were obtained with ampicillin containing liposomes [140]. Importantly, an enhanced splenic localization of the drug was demonstrated in these studies. Using a variety of nanoparticles antibacterial, antitubercular and antifungal drugs, such as doxycycline, econazole, ethionamide, gentamycin, moxifloxacin, streptomycin and rifampicin, have been intracellularly delivered in splenic macrophages [141,142,143,144,145,146,147,148]. A clinical condition that attracted much interest for a splenic macrophage targeting is the acquired immunodeficiency syndrome (AIDS) due to HIV-1 infection. Indeed, in this disease, viral accumulation and replication also take place in macrophages and macrophage-like cells that represent a reservoir for the virus and, in some cases, such as microglia in the brain, the primarily infected cell type. Therefore, targeting the macrophages may be helpful for the treatment of this disease. To this aim, several antiretroviral compounds have been targeted to macrophages also including splenic macrophages by incorporation in nanoparticles. For instance, Dutta and Jain [149] prepared dendrimers loaded with the nucleoside analogue reverse transcriptase inhibitor lamivudine and showed its selective macrophagic uptake in vitro, whereas Gajbhiye et al. [150] incorporated another member of this drug family, zidovudine, in dendrimers and studied its tissue distribution in rat. Interestingly, they found a very significant increase in zidovudine accumulation, not only in lymph nodes and in the lungs, but also in the spleen. By comparing different classes of dendrimers differing in their surface coating, including dendrimers coated with sialic acid (sialic acid conjugated polypropylenimine (PPI) dendrimers (SPPI)), with mannose (mannose conjugated PPI dendrimers (MPPI)) or with both sialic acid and mannose (sialic acid conjugated-mannosylated PPI dendrimers (SMPPI)), they concluded that both sialic acid and mannose receptors cooperate in macrophagic uptake of these nanoparticles with additive effects. Macrophagic accumulation and high disposition in the spleen have been demonstrated also for the protease inhibitors lopinavir loaded in modified pullulan nanoparticles [151] and for nanoformulated atazanavir [152].

In conclusion, splenic capture is a potentially major problem for the implementation of therapeutic uses of nanoparticles unless a splenotropic effect is desired. The use of stealth coating may minimize this problem, but, if PEG is used, the production of anti-PEG antibodies in the spleen may lead to the development of the ABC phenomenon and to the loss of stealth protection from splenic nanoparticle capture.

## 5. Role of the Spleen in the Pharmacokinetics of Exosomes

**Figure 5:**
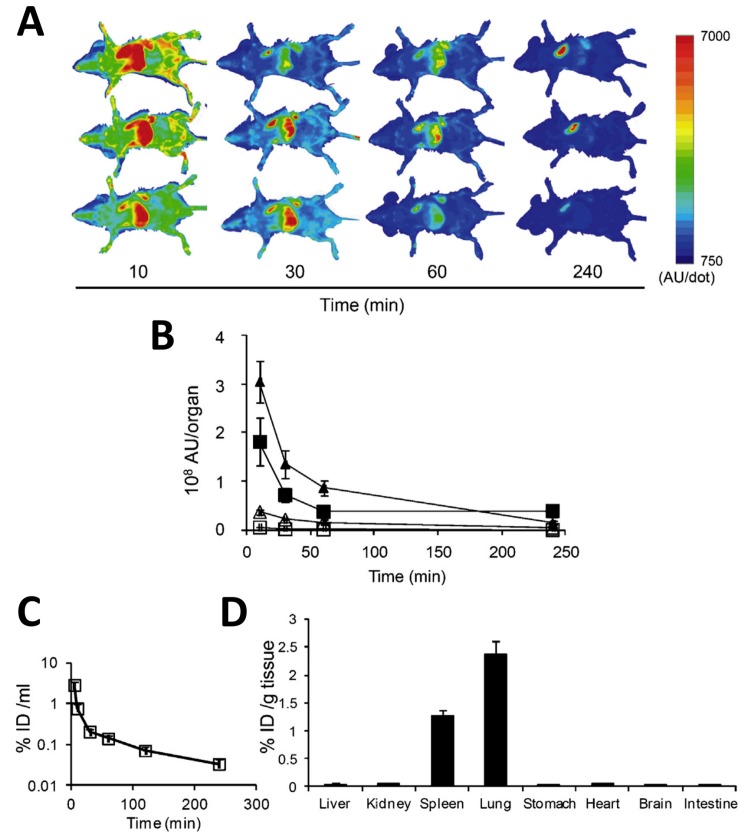
Tissue distribution of exosomes after intravenous administration in mice. (**A**) Tissue distribution of exosomes derived from luciferase expressing B16-BL6 melanoma cells visualized in vivo at different times after intravenous injection in mice. Exosomes were visualized by measuring bioluminescence emission by luciferase after injection of its substrate coelenterazine. The intensity in light emission is represented in pseudocolor; (**B**) Intensity of light emission by luciferase-expressing exosomes measured in arbitrary units in the liver (closed triangle), lung (closed square), kidney (open square) and spleen (open triangle) at different after intravenous injection; (**C**) Time course of luciferase activity in the serum measured as the percent of injected dose per mL (%ID/mL); (**D**) Exosome concentration measured in different organs as the percent of injected dose per gram of tissue four hours after the intravenous injection. Reproduced from [160] with permission from the publisher.

Exosomes are nanosized vesicular structures released by a variety of cells in physiological conditions or in disease states [153,154]. Despite the similar size range (30–100 nm), exosomes are profoundly different from liposomes. Indeed, they are not produced in the laboratory, but they are released from cells at the end of a complex biosynthetic process that involves the formation of multilamellar bodies inside endosomes and the sorting through a specific cargo machinery, the endosomal sorting complex required for transport (ESCRT) [155]. As such, exosomes have a complex lipid composition similar to plasma membranes, and they also contain integral proteins and glycoproteins some of which like the tetraspanins CD63, CD81 and CD9 are considered quite specific markers of these structures, and others, such as adhesion molecules and complement receptors, could be involved in their capture by the tissues [156]. Moreover, before being released from the cell, exosomes are loaded with signaling molecules, mainly miRNAs and proteins, that they will transport to distant target sites. This is because exosomes have physiological functions related to cell-cell communication. Exosomes that can be easily prepared by purification from the culture media of different types of donor cells, such as HEK293, HeLa or mesenchymal stem cells [156], attracted a considerable interest as tools for pharmacological interventions [157]. First and foremost, they can be loaded with pharmacologically-active compounds including not only siRNAs and miRNAs, but also conventional drugs, such as doxorubicin. The main advantage of using exosomes as drug delivery tools relies on the high efficiency of their internalization by target cells. In addition, exosomes may have interesting pharmacological activity by themselves as part of their intrinsic extracellular signaling physiological roles. For instance, specific exosomes have been demonstrated to boost the immune response to parasites, or to decrease new vessel formation in tumors, or to promote tissue regeneration in the infarcted heart [158]. Under the premises that exosomes could be used as pharmacological tools, the problem emerges of their in vivo disposition. Considering their similarity with liposomes and their size, which is relatively small and in a range in which liposome capture by the RES is minimized, it was originally supposed that tissue disposition would not be an issue. However, when pharmacokinetic studies were performed, it appeared clear that a significant portion of the administered exosomes does not reach their target tissues, because it is sequestered elsewhere, and that the spleen has an important role in this process. Sun et al. [159] evaluated the tissue distribution of fluorescent curcumin-loaded exosomes in mice. They found that as early as 1 h after the intraperitoneal injection of these vesicles, there was a significant accumulation of fluorescence in the live in the lung and in the spleen. However, a detailed pharmacokinetic analysis is precluded when using fluorescent exosomes because of sensitivity limitations. Therefore, alternative methods were developed to address this question. Takahashi et al. [160] transfected murine melanoma B16-BL6 cells with a plasmid encoding for a fusion protein made by Gaussia luciferase (gLuc), a reporter protein that emits light in the presence of its substrate coelenterazine, and lactadherin, an integral protein of the exosome membrane. The exosomes released by these transfected cells do express the fusion protein and will emit light in vitro in luciferase assays giving hence the opportunity to measure their concentration with high sensitivity. Using this approach, Takahashi et al. [160] showed that after being intravenously administered to mice, these luciferase-expressing exosomes disappeared from blood very quickly with a half-life of about 2 min because they were largely sequestered in tissues, mainly in the lungs and in the spleen (Figure 5). A preferential accumulation in the liver and in the spleen was also observed by Hwang et al. [161] who injected ^99m^Tc- hexamethylpropyleneamine oxime (HMPAO)-labeled exosomes into mice. The differential disposition of exosomes in tissues was measured by Morishita et al. [162] using ^125^l-labeled exosomes. More specifically, they collected the exosomes released by B16-BL6 cells transfected with constructs encoding for a fusion protein of streptavidin and lactadherin. Then, these exosomes were made radioactive by the incubation with ^125^l-labeled norbiotinamide and intravenously injected in mice. After 4 h 28%, 1.6% and 7% of the radioactivity was detected in the liver, spleen and lung, respectively.

Evidence was obtained that exosomes were mainly captured by macrophages because tissue sequestration was significantly reduced when the mice were pretreated with clodronate-containing liposomes, an experimental procedure that selectively depletes MZ macrophages in the spleen [163]. It has been demonstrated that phagocytosis is the principal mechanism of exosome internalization, and different mechanisms for exosome endocytosis in macrophages have been described [164,165]. A role in exosome capture has been identified for integrins and for lactadherin that binds to α_v_β_3_/β_5_ integrins and is expressed in exosomes from dendritic cells [166]. Exosome internalization by macrophages may also be induced by the deposition of fragments of the complement protein C3 on their surface [167], and evidence has been reported that the lectin galectin-5 is involved in macrophagic capture of red cell-derived exosomes [168]. Saunderson et al. [169] reported convincing evidence that exosomes could be captured by a specific subpopulation of macrophages, the CD169+ macrophages, via their interaction with the sialylated protein receptor CD169. CD169+ macrophages are selectively located in splenic MZ and in the subcapsular sinus and medulla of lymph nodes. Their physiological functions are still ill defined, although CD169+ macrophages could be involved in filtering particulate antigens entering the spleen or the lymph nodes, in antigen presentations or in the transfer of antigens to antigen-presenting cells [170]. The CD169 antigen that characterizes these cells belongs to the SIGLEC superfamily and binds sialylated proteins. Saunderson et al. [169] showed that the main ligand of this receptor, α2,3-linked sialic acid, is highly expressed in the exosome membrane and that exosome disposition is markedly altered in CD169 knockout mice as compared with their wild-type littermates. Indeed, although exosomes showed a similar circulating half-life in these two groups of animals, in CD169 knock-out mice, they penetrated deeper regions with respect to wild-type controls both in the lymph nodes and in the spleen where they reached the outer marginal zone and the red pulp. Importantly, the immunologic response to antigen pulsed exosomes was also enhanced in CD169 knockout mice in comparison with wild-type mice, hence suggesting that upon sequestering by CD169 macrophages, exosomes become ineffective and are probably destroyed.

In conclusion, the spleen is a key anatomical station for miRNA and exosome sequestration, and a thorough understanding of the mechanisms involved in this process will be demanded to develop modified versions of these vesicles with the final aim of escaping their splenic capture and increasing their biological activity.

## 6. General Conclusions

The data that we reviewed in the present paper clearly indicate that because of its anatomical characteristics, such as its high blood flow and loose capillaries, the spleen is highly exposed to circulating drugs. Whereas it is unlikely that the spleen could significantly impact systemic disposition of classical drugs, splenic capture of new generation drugs may have important implications by different mechanisms. More specifically, monoclonal antibodies directed against antigens expressed in the spleen directly affect the function of this organ, whereas drug-conjugated monoclonal antibodies exert a local toxic effect upon release of their conjugated chemotherapeutic drugs. Moreover, the spleen represents an important clearance site for exosomes and nanoparticles and may direct immune responses against their PEG stealth. In conclusion, the spleen, this small, neglected organ, continues to surprise us with unexpected physiological roles: after having been recently linked to the pathophysiology of non-alcoholic fatty liver disease [171], a new role is emerging for the spleen in the pharmacokinetics of new generation drugs with possible relevant implications on their efficacy and toxicity, and this small, neglected organ will certainly deserve stronger attention by pharmacologists in the future.
